# COVID-19 Vaccine Hesitancy in Italian Adults with Marfan Syndrome: Insights from a Secondary Analysis of a Cross-Sectional Study

**DOI:** 10.3390/vaccines11040734

**Published:** 2023-03-26

**Authors:** Nathasha Samali Udugampolage, Alessandro Pini, Arianna Magon, Gianluca Conte, Edward Callus, Jacopo Taurino, Rosario Caruso

**Affiliations:** 1 Cardiovascular-Genetic Center, IRCCS Policlinico San Donato, 20097 Milan, Italy; 2 Health Professions Research and Development Unit, IRCCS Policlinico San Donato, 20097 Milan, Italy; 3 Department of Biomedical Sciences for Health, University of Milan, 20133 Milan, Italy; 4 Clinical Psychology Service, IRCCS Policlinico San Donato, 20097 Milan, Italy

**Keywords:** attitudes, beliefs, clinical factors, COVID-19, cross-sectional study, Italy, Marfan syndrome, mental health, sociodemographic factors, vaccine hesitancy

## Abstract

Although vaccine hesitancy has been reported in many patient groups and countries, there is a lack of data on vaccine hesitancy in patients with Marfan syndrome (MFS). MFS is a rare genetic disorder that can lead to cardiovascular, ocular, and musculoskeletal issues. Because MFS patients may face an increased risk of COVID-19 complications, vaccination is crucial for this population. This brief report aims to describe vaccine hesitancy rates in MFS patients and compare the characteristics of patients who are hesitant and those who are not to gain a better understanding of this specific population. This study analyzes previously published cross-sectional data that examined mental health, sociodemographic, and clinical factors associated with PTSD, depression, anxiety, and insomnia in MFS patients during the third wave of the COVID-19 pandemic in Lombardy, Italy. Of the 112 MFS patients who participated, 26 (23.9%) reported vaccine hesitancy. Vaccine hesitancy may be associated mainly with younger age and not be related to other patient characteristics. Therefore, this report found no differences in individual-level variables, such as sex, education, comorbidities, and mental health symptoms, between those who were hesitant and those who were not. The study findings are insightful and suggest that interventions to address vaccine hesitancy in this population may need to focus on attitudes and beliefs related to vaccination rather than targeting specific sociodemographic or clinical factors.

## 1. Introduction

COVID-19 vaccine hesitancy has become a significant public health concern, as widespread vaccination is crucial for ending the COVID-19 pandemic and returning to normalcy [1,2,3,4,5]. COVID-19 vaccine hesitancy refers to the reluctance or unwillingness of individuals to receive a COVID-19 vaccine, despite its availability and recommendation by healthcare professionals and public health organizations [6]. Vaccine hesitancy can have several dimensions, including concerns about the safety and effectiveness of the vaccine, mistrust of the healthcare system or government, religious or cultural beliefs, fear of side effects, and misinformation or misconceptions about the vaccine [1,2,3,4,5,6]. Although vaccine hesitancy was described in several groups of patients [7,8,9], the general public [5,10], and in several countries [7,8,9,10,11], the available information on COVID-19 vaccine hesitancy is currently lacking in specific subgroups of patients, as per adults with Marfan syndrome (MFS) [12].

MFS is a rare autosomal disorder that can cause a range of symptoms, including cardiovascular, ocular, and musculoskeletal manifestations [13]. Prompt diagnosis is crucial for providing the best treatment to patients. In this regard, the diagnosis of the syndrome relies on a systemic score calculation and DNA mutation identification [14,15]. A multidisciplinary team is required to manage the potentially life-threatening complications of MFS, from the comprehensive characterization of the disease to the treatment and management [16,17,18,19]. Considering the cardiovascular involvement in MFS in several phenotypes, these patients may be at an increased risk of complications from COVID-19, as per other patients with underlying diseases. This makes vaccination especially important for this population, as it can help protect them from COVID-19 and its potentially severe effects [12,15]. Furthermore, knowing the rates of vaccine hesitancy among patients with MFS is important to allow healthcare providers to create tailored strategies and messages to address the specific concerns or issues contributing to vaccine hesitancy.

Thus far, there are no specific data regarding COVID-19 vaccine hesitancy in patients with MFS, even in the context of previous descriptions regarding the general acceptance of the COVID-19 vaccine in the general Italian population and its related factors [20,21]. At the end of 2020, the acceptance of COVID-19 vaccination among older adults in southern Italy showed that the majority of enrolled patients were willing to be vaccinated, with higher acceptance associated with higher education levels and reliance on social/mass media for information, while the introduction of the green pass was negatively associated with acceptance [21], where the green pass was a digital certificate issued by the Italian government that provided proof of vaccination against COVID-19, a negative test result, or recovery from COVID-19. Another study evaluated the knowledge and acceptance of COVID-19 vaccination in Italian undergraduate students and found that 91.9% of participants were willing to receive the vaccine, with correct knowledge related to acceptance, indicating the effectiveness of communication strategies accompanying the COVID-19 immunization campaign in Italy [20]. In the context of this literature, the lack of specific data regarding COVID-19 vaccine hesitancy in patients with MFS is concerning. It may lead to inadequate vaccine uptake and hinder efforts of general untargeted vaccine campaigns. Without understanding the rate of vaccine hesitancy and its contributing factors in this population, it may be difficult to develop targeted strategies and messages to address concerns and encourage vaccination. For this reason, this brief report described vaccine hesitancy rates in patients with MFS and focused on comparing individual-level characteristics of patients with and without COVID-19 vaccine hesitancy to boost insights for this specific population.

## 2. Materials and Methods

### 2.1. Design

This brief report is a secondary analysis of a previously published cross-sectional study [12]. A secondary analysis from a cross-sectional study involves using the data collected in the original cross-sectional study for a different research question or objective. In other words, we analyzed the existing data to answer a new research question that was not addressed in the original study [22]. Precisely, the original cross-sectional study aimed to evaluate post-traumatic stress disorder (PTSD), depression, anxiety, and insomnia in patients with MFS during the third wave of the COVID-19 pandemic (between February and April 2021) in a region of northern Italy (Lombardy) and determine which mental health, sociodemographic, and clinical factors were associated with PTSD [12]. This type of analysis can be useful for maximizing the value of existing data and resources, especially in relation to rare diseases as per MFS, and when conducting a new study is not feasible or practical. The previously published study used a single-center and convenience sampling approach [22]. The authors used one proportion from a finite population approach to estimate a sample size that would represent patients with MFS living in Lombardy, Italy. To determine the appropriate sample size for the study, the researchers used a formula that accounted for the population size of roughly 1500 patients in Lombardy. The formula included a variable, X, which was determined by a few factors: the sample proportion of 8%, which was the rate of mental health scores using the 12-item Short Form Survey under the third quartile of a previous study [17] and the margin of error set to 5%. The formula was X/(X + population size−1) = Z_α/2_^2^*(1 − sample proportion)/(margin of error). The required minimum sample size for a 95% confidence interval and a sample proportion of 8% (the rate of mental health patients under the 75th percentile) was, therefore, 106 patients. In other words, even if the sample of 112 enrolled patients in the analysis is limited, it was adequate to represent the population living in Lombardy based on this estimate.

### 2.2. Source of Data

The raw data from a previously published study [12] were used to describe vaccine hesitancy rates in patients with MFS and compare individual-level characteristics of patients with and without COVID-19 vaccine hesitancy to boost insights for this specific population. For this brief report, the variables included in the original study were all included.

The study was conducted in an MFS-specialized center in Lombardy, Italy, between February and April 2021, during the third wave of the COVID-19 pandemic, using a descriptive observational design with cross-sectional data collection. Patients were asked to fill out validated questionnaires that assessed PTSD, depression, anxiety, and insomnia and provided sociodemographic, clinical, and anamnestic data. A total of 112 patients with MFS out of 154 eligible and invited patients agreed to participate in the study, and all 112 completed the questionnaires. In addition, a question regarding COVID-19 vaccine hesitancy was included in line with previous questions aimed at investigating this phenomenon. A total of 26 patients (23.9%) out of 112 reported COVID-19 vaccine hesitancy.

The study found that patients with COVID-19 had high rates of psychological symptoms of depression, anxiety, and insomnia, as well as post-traumatic stress disorder (PTSD). About 20.5% of patients reported clinical concern for PTSD. Female patients, older patients, and non-active workers reported higher scores of PTSD, while patients without respiratory or other comorbidities reported lower scores of PTSD. Patients who previously had psychotherapy or had been prescribed psychoactive drugs had higher PTSD scores. Anxiety was the strongest predictor of PTSD scores. Older patients and non-active workers also had higher scores for avoidance, and patients prescribed psychoactive drugs had lower scores for avoidance.

### 2.3. Variables and Data Analysis

Data from the original dataset were analyzed by comparing each individual-level variable between the subgroup of patients with and without vaccine hesitancy with nonparametric statistics. More precisely, the available variables were sex (males, females, other), education (lower than secondary school, high school diploma, university), occupation (active workers, unemployed, or retired), age (years), time from diagnosis (years), cardiovascular comorbidities (yes, no), previous abdominal surgery (yes, no), in treatment with cardiovascular medications (yes, no), respiratory comorbidities (yes, no), other comorbidities, previous psychotherapy (yes, no), psychiatric or psychological support during the COVID-19 pandemic (yes, no), prescribed psychoactive drugs (yes, no), having reported at least one positive test for the COVID-19 (yes, no), having downloaded the Italian tracking system (IMMUNI) (yes, no), concerns about being infected (yes, no), Impact of Event Scale-Revised (IES-R) [23], depression with the Patient Health Questionnaire-9 (PHQ-9) [24], anxiety with the General Anxiety Disorder-7 (GAD-7) [25], and Insomnia Severity Index (ISI) [26]. Vaccine hesitancy was assessed as the total unwillingness or strong preference to avoid the COVID-19 vaccine (situations were combined) [10].

Categorical variables were compared by employing a chi-squared test or Fisher’s exact test if appropriate. Scores were compared by using the Mann–Whitney U test. Missing data (lower than 3%) were managed with an available case approach without imputations. In this analytical stage, as multiple comparisons were planned, Bonferroni correction was applied to determine adequate probabilistic thresholding to mitigate the likelihood of false positive inferential results [27]. Therefore, the significance level for the multiple comparisons was α/k, where α is 0.05 and k (i.e., number of comparisons) is 21, implying an adjusted α equal to 0.002.

The individual-level variables showing a trend that suggests descriptive differences between hesitant and non-hesitant patients were selected as predictors of a logistic regression model having vaccine hesitancy as the outcome (i.e., vaccine hesitancy: yes vs. no). The estimated associations between predictors and vaccine hesitancy were reported as odds ratios (ORs) with 95% confidence intervals (CIs). The goodness of fit of the logistic regression model was assessed using the Hosmer–Lemeshow test and a pseudo-*R*^2^ statistic. The Hosmer–Lemeshow test assessed whether the observed and expected frequencies of the outcome variable are similar across different groups defined by the predicted probabilities. A non-significant result indicates that the model fits well, while a significant result indicates a poor fit. Nagelkerke’s pseudo-*R*^2^ statistic measured the proportion of variance in the outcome variable explained by the model, with higher values indicating a better fit. As the employed predictors in the model were “age” (years) and “at least one previous positive COVID-19 test” (yes vs. no), a plot was developed where the *x*-axis represented the age (years), and the *y*-axis represented the predicted probabilities of the vaccine hesitancy taking on the value of “yes”. The plot illustrated the associations between “previous positive COVID-19 test (at least one: yes vs. no)” and vaccine hesitancy (yes). The associations that were derived from the previous positive COVID-19 test were partitioned to show the changes in the ORs (and their 95% CIs) of having a previous positive COVID-19 test or not, in association with vaccine hesitancy and in relation to age. This approach was employed to allow researchers an in-depth interpretation of the estimates derived from the model. Null hypotheses were two-sided, and analyses were performed, setting alfa = 5% and using Stata Statistical Software version 17.0 (College Station, TX, USA: StataCorp LP).

### 2.4. Ethical Considerations

The Ospedale San Raffaele Institutional Review Board approved the study protocol (project COGEAD, protocol number: 01/02/2021). Each participant provided their full and informed consent electronically before enrolling in the study. The research methods were in accordance with ethical standards, Good Clinical Practice guidelines, and the International Council for Harmonization of Technical Requirements for Pharmaceuticals for Human Use.

## 3. Results

**Figure 1:**
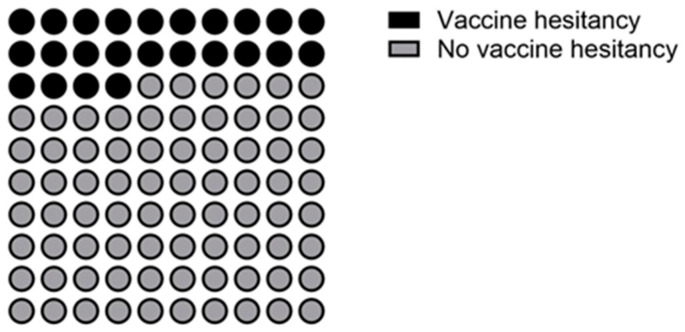
Distribution of vaccine hesitancy in adults with MFS (n = 112).

Figure 1 shows the distribution of COVID-19 vaccine hesitancy (23.9%).

**Figure 2:**
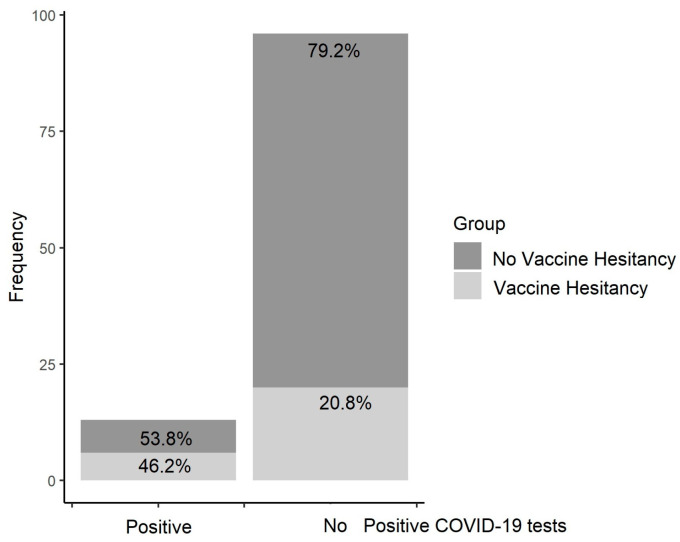
Vaccine hesitancy in patients with and without previous positive COVID-19 test.

The comparisons of the characteristics of patients with and without vaccine hesitancy are shown in Table 1. The only two comparisons that highlighted a descriptive trend suggesting differences were related to the rates of a previous positive test for COVID-19 and age. As Figure 2 depicts, in the group of patients who reported at least one previous positive COVID-19 test, the rates of patients with vaccine hesitancy (46.2%) tended to be higher than those (20.8%) who had not reported any previous positive COVID-19 test (*p* = 0.044) (see Figure 2).

**Figure 3:**
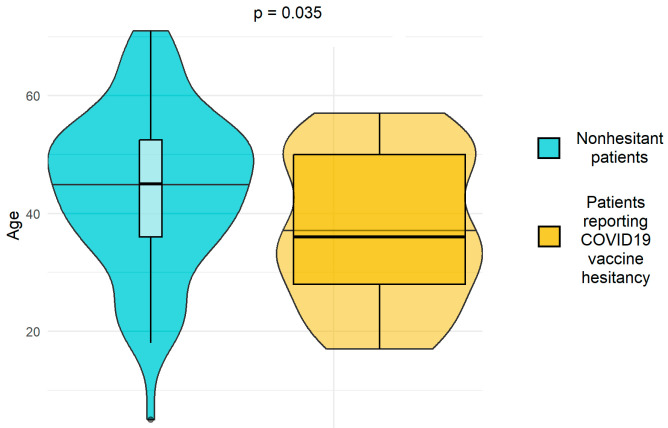
Age distribution between non-hesitant and hesitant patients.

As shown in Figure 3, patients reporting COVID-19 vaccine hesitancy tended to be younger than non-hesitant patients (*p* = 0.035).

As shown in Table 2, the logistic regression model testing the relationships between age and previous positive COVID-19 with vaccine hesitancy showed adequate fit to explain the sample statistics (χ^2^_(8)_ = 6.756; *p* = 0.563). Each additional year in the age of the enrolled patients was significantly associated with a reduction of 3.6% in the odds of reporting vaccine hesitancy (OR = 0.964; 95% CI = 0.931–0.998; *p* = 0.036). Having at least one previous positive COVID-19 test tended to increase by approximately three times the odds of reporting vaccine hesitancy (OR = 3.338; 95% CI = 0.995–11.24; *p* = 0.052).

**Figure 4:**
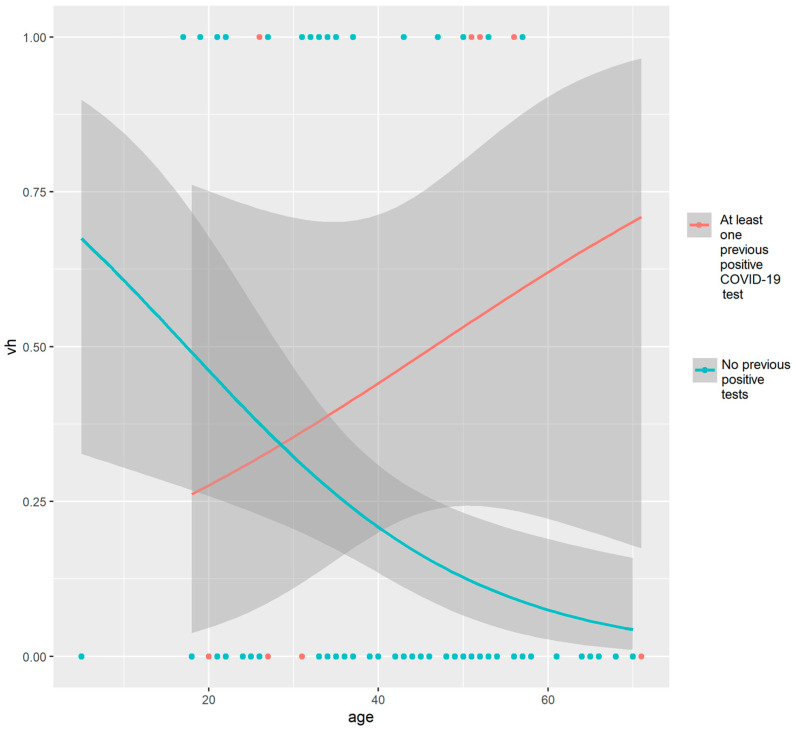
Associations between vaccine hesitancy (vh) and having or not having previous COVID-19 test results in relation to the age of patients.

By plotting the OR curves (and their 95% CIs) over age categories, the associations between vaccine hesitancy and having or not having previous COVID-19 test results were examined. The analysis revealed that younger patients who did not report a previous positive COVID-19 test had a higher likelihood of vaccine hesitancy, while the odds decreased among older patients (with a narrower 95% CI). On the other hand, the group with at least one previous positive COVID-19 test reported increased ORs among patients over 40 years of age (see Figure 4).

## 4. Discussion

This report showed that in a cohort of 109 patients with MFS, 23.9% reported vaccine hesitancy related to COVID-19. There were no significant associations between vaccine hesitancy and any patient characteristics, even though a trend of descriptive differences emerged for the rates of previous infections and age. It is possible that hesitant patients of higher age were those that do not want the COVID-19 vaccine even if they experienced COVID-19, while the hesitation in the younger population was mainly among those who had not experienced COVID-19. However, this is only a possible explanation, and further research is needed to confirm this hypothesis.

The interpretation of these results should consider the study’s relatively small sample size for performing inferential tests (even if the logistic regression was well-fitted to sample statistics). In addition, it is important to consider the epidemiological distribution of positive COVID-19 test results during the third wave of the pandemic in Italy, which primarily affected individuals over the age of forty [28]. This distribution may have impacted the study’s findings, and therefore, additional research with larger sample sizes and across various geographic regions may be necessary to validate and generalize these results.

Understanding the factors contributing to vaccine hesitancy in patients with MFS can provide insight into addressing vaccine hesitancy in this population and improving vaccination rates. This aspect is meaningful for the increased risk of complications that patients with MFS might have if infected with severe COVID-19 manifestations. Previous research in different Italian populations has shown that vaccine hesitancy is a complex phenomenon influenced by various factors such as social and cultural beliefs, access to information, and trust in healthcare providers [26], even if no data regarding patients with MFS are available in relation to the COVID-19 vaccine hesitancy. For instance, two studies conducted in Italy at the end of 2020 and early 2021 assessed acceptance and knowledge of COVID-19 vaccination among older adults and university students, revealing high acceptance rates (higher than 90%) and good levels of knowledge, respectively, indicating the effectiveness of communication strategies accompanying the COVID-19 immunization campaign in Italy [20,21]. In the current study, the lack of significant associations between vaccine hesitancy and patient characteristics should be interpreted with caution as the sample size for those with a positive COVID-19 test was small (n = 13), and the difference in rates of positive tests between the two groups was relatively small. This result implies that vaccine hesitancy in this population may not be related to any particular sociodemographic or clinical factors except for age and previous positive COVID-19 tests. Instead, vaccine hesitancy may reflect a tendency toward general reluctance to receive vaccinations [29,30].

Based on analyzed data, it seems that vaccine hesitancy in patients with MFS may have similar characteristics to vaccine hesitancy in the general population [29,31], even if data on the general Italian population collected at the end of 2020 suggest a higher acceptance rate among participants enrolled in previous studies [20,21]. However, it is important to note that this study only included a small sample size of 112 individuals, and the findings may not be representative of the broader population of patients with MFS, even if considering that MFS is a rare disease, this sample is worthy of being considered insightful (its prevalence is estimated to be around 1 in 5000–10,000 individuals worldwide). In fact, vaccine hesitancy in this report was not significantly associated with sex, education, occupation, presence of comorbidities, previous psychotherapy, use of psychoactive drugs, or COVID-19 tracking system usage. These findings suggest that interventions to address vaccine hesitancy in people with MFS may need to focus on attitudes and beliefs related to vaccination rather than targeting specific sociodemographic or clinical factors.

The emerging evidence that vaccine hesitancy in patients with MFS has similar characteristics as the vaccine hesitancy previously described in the Italian and international population means that strategies for mitigating hesitancy were weak for this specific subgroup of patients even if patients with chronic conditions are typically more engaged in lifelong medical treatments [29,31]. This aspect emphasizes the fact that vaccine hesitancy in patients with chronic conditions was a concern even before COVID-19, where patients with potential high trust in biomedical research, such as those that periodically attend follow-up and take medications as per patients with MFS, do not have better features of vaccine hesitancy than the ones of the general population and might even report slightly lower acceptance rates compared with the general Italian population [20,21].

In Italy, previous research has shown the sociodemographic, psychological, belief, and behavioral characteristics of three groups: accepters, rejecters, and fence sitters (those that have to decide) [32]. The fence sitters group included individuals of younger age, lower educational level, and not a stable economic situation. Factors associated with being a fence sitter rather than a vaccine accepter or rejecter included lower levels of protective behaviors, trust in institutions and informational sources, frequency of use of informational sources, agreement with restrictions, and higher conspirative mentality. Previous evidence from the general Italian population has demonstrated that trust in the scientific community was the strongest predictor of vaccine acceptance (not only in relation to COVID-19) [33]. To frame the understanding of how Italians perceived the COVID-19 vaccination campaign, a study compared Italian language tweets before and after the COVID-19 vaccination campaign, revealing polarization and volumes of tweets with a potential impact on vaccine hesitancy: 29.6% of users as anti-Vax and 12.1% as pro-Vax, with a change in retweets after the start of the campaign [34]. In general, rates of vaccine hesitancy ranged between 14% and 35%, with variations associated with age and periods of data collection [7,10]. Other studies showed vaccine hesitancy rates lower than 10% among older adults and university students, with a tendency of higher vaccine hesitancy in people with poor acceptance of the adoption of the digital certificate issued by the Italian government that provided proof of vaccination against COVID-19, a negative test result, or recovery from COVID-19 (i.e., green pass) [20,21].

In general, the 5C model of the drivers of vaccine hesitancy can be used to identify five main reasons for vaccine hesitancy, which are confidence in the vaccine, complacency about the disease, convenience of getting vaccinated, calculation of personal risk, and collective responsibility for public health [35]. For instance, concerns about vaccine side effects were the most common reasons for hesitancy among the patients with MFS in this study, and they relied on healthcare workers as the most trusted sources of guidance. However, misinformation and incomplete information on the internet and social media can also pose challenges in making informed decisions about vaccination. Therefore, an in-depth focus on vaccine hesitancy using the 5C model should be performed in future studies to tailor interventions that address the specific concerns and attitudes of patients with MFS.

This report has several limitations that must be acknowledged and are implicit in the nature of a secondary analysis of a previously published study. The performed analyses had limited control over the design and methods used in the original study, which may not align with their specific research question. In fact, elements regarding confidence, complacency, convenience, risk calculation, and collective responsibility would be pivotal in original research to investigate vaccine hesitancy and, in this study, were not available.

## 5. Conclusions

This report suggests that vaccine hesitancy in patients with MFS may be associated mainly with younger age and not be related to other particular sociodemographic or clinical factors but instead may reflect a general reluctance to receive vaccinations. Although limited by the small sample size, the study findings are insightful and indicate that interventions to address vaccine hesitancy in this population may need to focus on attitudes and beliefs related to vaccination rather than targeting specific sociodemographic or clinical factors. The report highlights the need for a more in-depth focus on vaccine hesitancy in patients with MFS to identify the main reasons for vaccine hesitancy and provides important insights into vaccine hesitancy in a rare disease population and could help inform future interventions to increase vaccine uptake in this population.

## Figures and Tables

**Table 1:** Characteristics of the responders (n = 112).

		Vaccine Hesitancy (n = 26; 23.9%)		No Vaccine Hesitancy (n = 83; 76.1%)		*p*
		N	%	N	%	
Sex						
	Males	13	50.0	32	38.6	0.301
	Females	13	50.0	51	61.4	
Education						
	Lower than secondary	8	30.8	19	22.9	0.719
	High school diploma	11	42.3	39	47	
	University	7	26.9	25	30.1	
Occupation						
	Active worker	15	57.7	50	60.2	0.817
	No active worker	11	42.3	33	39.8	
Age						
	Years (median; IQR)	36	26.7–50.3	45	36.0–53.0	0.035
Years from diagnosis						
	Years (median; IQR)	10	5.0–20.0	15	7.0–22.0	0.387
Cardiovascular comorbidities						
	Yes	18	69.2	69	83.1	0.123
Previous abdominal surgeries						
	Yes	9	34.6	38	45.8	0.316
In treatment with cardiovascular drugs						
	Yes	17	65.4	61	73.5	0.424
Respiratory comorbidities						
	Yes	6	23.1	13	15.7	0.385
Other comorbidities						
	None	26	100.0	78	94.0	0.440
	Thyroid			3	3.6	
	Neuropathies			2	2.4	
Previous psychotherapy						
	Yes	8	30.8	23	27.7	0.763
Psychiatric or psychological support during the COVID-19 pandemic						
	Yes	4	15.4	10	12	0.657
Prescribed psychoactive drugs						
	Yes	4	15.4	15	18.1	0.753
Prescribed psychoactive drugs during the pandemic						
	Yes	1	5	5	7.8	0.670
Having reported at least one positive test for COVID-19						
	Yes	6	23.1	7	8.4	0.044
Having downloaded the Italian tracking system (IMMUNI)						
	Yes	4	15.4	26	31.3	0.112
Great concerns about being infected by COVID-19						
	Yes	7	26.9	36	43.4	0.134
PHQ-9						
	Score (median; IQR)	7	3–11	5	3–9	0.539
GAD-7						
	Score (median; IQR)	5.5	1–9.25	5	2–8	0.833
Insomnia						
	Score (median; IQR)	6	2.75–12	5	2–11	0.615
Avoidance						
	Score (median; IQR)	3	0–10	4	0–9	0.678
Intrusion						
	Score (median; IQR)	2.5	0–10.25	4	1–8	0.830
Hyperarousal						
	Score (median; IQR)	1	0–5.5	1	0–5	0.705
Total IES-R						
	Score (median; IQR)	8.5	0.75–25.75	11	2–21	0.773

Legend: IQR—interquartile range; PHQ-9—Patient Health Questionnaire-9; GAD-7—anxiety with the General Anxiety Disorder-7; IES-R—Impact of Event Scale-Revised; avoidance, intrusion, and hyperarousal are specific domains of the IES-R scale. Note: significant differences require *p* < adjusted α (i.e., 0.002).

**Table 2:** Logistic regression model testing the relationships between age and previous positive COVID-19 with vaccine hesitancy (yes vs. no).

	OR	95% CI	*p*
Age (years)	0.964	0.931–0.998	0.036
At least one positive COVID-19 test	3.338	0.995–11.24	0.052
Pseudo-*R*^2^	0.13		
Hosmer–Lemeshow test	χ^2^_(8)_ = 6.756; *p* = 0.563		

## Data Availability

Data are available from the corresponding author (R.C.) upon reasonable request.
